# Dihydroartemisinin-Transferrin Adducts Enhance TRAIL-Induced Apoptosis in Triple-Negative Breast Cancer in a P53-Independent and ROS-Dependent Manner

**DOI:** 10.3389/fonc.2021.789336

**Published:** 2022-01-03

**Authors:** Xinyu Zhou, Abel Soto-Gamez, Fleur Nijdam, Rita Setroikromo, Wim J. Quax

**Affiliations:** ^1^ Department of Chemical and Pharmaceutical Biology, Groningen Research Institute of Pharmacy, University of Groningen, Groningen, Netherlands; ^2^ European Institute for the Biology of Aging (ERIBA), University Medical Center Groningen (UMCG), Groningen, Netherlands

**Keywords:** triple-negative breast cancer (TNBC), dihydroartemisinin (DHA), transferrin (TF), TRAIL-induced apoptosis, death receptor (DR)

## Abstract

Triple-negative breast cancer (TNBC) is a highly aggressive breast cancer subtype independent of estrogen receptor, progesterone receptor, or human epidermal growth factor receptor 2. It has a poor prognosis and high recurrence. Due to its limited treatment options in the clinic, novel therapies are urgently needed. Single treatment with the death receptor ligand TRAIL was shown to be poorly effective. Recently, we have shown that artemisinin derivatives enhance TRAIL-induced apoptosis in colon cancer cells. Here, we utilized transferrin (TF) to enhance the effectiveness of dihydroartemisinin (DHA) in inducing cell death in TNBC cell lines (MDA-MB-231, MDA-MB-436, MDA-MB-468 and BT549). We found that the combination of DHA-TF and the death receptor 5-specific TRAIL variant DHER leads to an increase in DR5 expression in all four TNBC cell lines, while higher cytotoxicity was observed in MDA-MB-231, and MDA-MB-436. All the data point to the finding that DHA-TF stimulates cell death in TNBC cells, while the combination of DHA-TF with TRAIL variants will trigger more cell death in TRAIL-sensitive cells. Overall, DHA-TF in combination with TRAIL variants represents a potential novel combination therapy for triple-negative breast cancer.

## Introduction

Breast cancer is the most prevalent malignant carcinoma in women who were diagnosed with cancer worldwide. Endocrine therapy targeting estrogen receptor or progesterone receptor is a well-tolerated treatment for some breast cancer patients (1). However, about 15-20% of breast cancers are triple-negative breast cancer (TNBC), which is known as a subtype lacking estrogen receptor, progesterone receptor, or human epidermal growth factor receptor 2 (2, 3). As a result, TNBC is resistant to endocrine therapy and shows high metastasis and poor prognosis with a low median survival time (4). Thus, there is an urgent demand for an effective, low-cost, and easy to apply therapy for TNBC.

It has been documented that artemisinin (ART) and its semi-synthetic derivatives are effective in treating cancers (5–7), including breast carcinomas (8, 9). They are sesquiterpene lactone compounds used as standard drugs in treating malaria. Compared with artemisinin and some derivatives, dihydroartemisinin (DHA) exhibits higher efficacy, solubility, and bioavailability as a therapeutic agent (10, 11). In malaria, parasites digest hemoglobin resulting in an iron-rich internal environment essential for the activation of artemisinins (12). The low-valent transition irons cleave the endoperoxide bridge of artemisinins generating intraparasitic carbon-centered free radicals or reactive oxygen species (ROS) and ultimately kill the *Plasmodium falciparum* (13, 14). Intriguingly, artemisinins also shows a specific cancer-killing nature due to the iron-rich environment present in proliferating cancer cells. However, compared to other breast cancers or other tumors in TNBC, artemisinins provide a relatively weak cell killing activity. However, its anti-cancer activity can be enhanced by forming drug-protein adducts with transferrin (TF) as was demonstrated in lung and liver cancer cells (15). Hence, it is meaningful to utilize both DHA and TF in TNBC cells and investigate the efficacy.

Human transferrin is a blood-plasma glycoprotein, which binds and transports iron in the human body to maintain iron homeostasis and metabolism. Iron-saturated holo-transferrin (holo-TF) transports irons into cells and releases irons in the acidic endosome to accomplish intracellular iron delivery (16). Using fluorescence spectra and UV–vis absorption assays, it was established that DHA binds to transferrin *via* hydrophobic and van der Waals interactions (15, 17). The DHA-TF adducts are formed with high binding affinity in neutral environments, allowing transferrin to transport DHA into cells by transferrin receptor (TfR)-mediated endocytosis. Subsequently, the decreased binding capacity at acidic conditions in endosomes leads to the dissociation of DHA and iron from transferrin, which activates DHA to form ROS in cancer cells (15, 18). Thus, it is of interest to evaluate the effect of DHA-TF adducts in TNBC cells for stimulating ROS production and inducing cell death.

It has been established that artemisinin’s anti-cancer mechanism acts mainly through the cleavage of the endoperoxide bridge, which eventually induces cell ferroptosis, apoptosis, autophagy, cell cycle arrest, or anti-angiogenesis (19). ROS, which are highly reactive chemical molecules formed by losing one oxygen electron from the cleaved endoperoxide bridge, are essential to the above-mentioned physiological processes (20, 21). There are multiple ROS species, including superoxide $\left({\text{O}}_{2}^{-}\right)$ , hydrogen peroxide (H_2_O_2_), hydroxyl radicals (·OH), peroxide $\left({\text{O}}_{2}^{2-}\right)$ , and hydroxyl ion (OH^-^). Many studies have shown that artemisinins produce ROS in parasites and cancer cells, while Egwu et al. reported specifically that superoxide is generated when parasites are treated with artemisinins (22). We, therefore, analyzed the production of both ROS and superoxide from DHA or DHA-TF within TNBC.

To further establish the efficacy of DHA-TF on TNBC, we became interested in combining it with tumor necrosis factor-related apoptosis-inducing ligand (TRAIL). TRAIL-induced apoptosis is an irreversible programmed cell death initiating intrinsic pathway and/or extrinsic pathway (23). Trimerized TRAIL binds to the extracellular domain of death receptor 4 (DR4) or death receptor 5 (DR5) to stimulate caspase cascade activation, eventually leading to cell shrinkage, DNA fragmentation, and cell apoptosis (24). TRAIL is harmless to non-transformed cells and our previous research on colon cancer cells showed that DHA enhances TRAIL efficacy, especially the DR5-specific TRAIL variant DHER-induced apoptosis (25).

In this study we found that the treatment of DHA-TF adducts perform a better efficacy than individual DHA in TNBC. It also results in DR5 upregulation and thereby enhances DHER-induced apoptosis. Above all, we confirm that the DHA-TF combination with DHER shows a promising medication effect in TNBC cell lines making it a potential therapeutic for TNBC in the future.

## Materials and Methods

### Cell Lines and Reagents

The breast cancer cell lines MDA-MB-231, MDA-MB-436 and MDA-MB-468 were cultured in DMEM medium and BT-549 was cultured in RPMI-1640 medium in a humidified incubator at 37°C and 5% CO_2_. All the cell lines were obtained from American Type Culture Collection (ATCC, Wesel, Germany). Media contain 10% fetal bovine serum (FBS, Costar Europe, Badhoevedorp, Netherlands) supplemented with 100 units/mL penicillin, and 100 µg/mL streptomycin (Gibco, Bleiswijk, Netherlands). The wild type (WT) TRAIL, DR4-specific TRAIL (4C7) and DR5-specific TRAIL (DHER) were produced as described (26, 27). Dihydroartemisinin (DHA; Adooq Bioscience, Irvine, CA, USA) stocks were dissolved in dimethyl sulfoxide (DMSO) to 10 mM, and the DMSO concentrations were less than 1% in all treatments. Holo-Transferrin (TF; Sigma-aldrich, St. Louis, USA) was dissolved in PBS to a final concentration of 0.5 mM. Pfithrin alpha, Q-VD-Oph, liproxstatin-1 (Bio-Connect B.V., Netherlands), ferrostatin-1 (Adooq Bioscience, Irvine, CA, USA) were dissolved in DMSO. N-Acetyl-L-cysteine (Sigma-aldrich, St. Louis, USA) was prepared to a final concentration of 600 mM in PBS, pH 7.4.

### The Formation of DHA-TF Adducts

Fluorescence emission spectra were recorded by a BioTek™ Synergy™ H1 Hybrid Multi-Mode Fluorescence Microplate Reader (Biotek, Winooski, VT, USA) using UV-STAR microplates (Greiner bio-one, Alphen a/d Rijn, Netherlands). Different concentrations of transferrin or DHA were diluted in PBS (pH 7.4). Fluorescence spectra were recorded from 300 nm to 400 nm at room temperature (RT) (λex=270 nm) (15, 17). The DHA was added into TF solution and incubated for 30 min. to perform the measurements. For the continued analysis on cells, the DHA and TF were diluted in medium (without FBS) with a 1:1 molar ratio and incubate at RT for 30 min. to form adducts.

### Cell Viability Assay

MDA-MB-231, MDA-MB-436, MDA-MB-468 and BT-549 were seeded in triplicate in 96-well plates at cell densities of 10,000 cells/well for 24h. Then DHA, TF, DHA-TF adducts with gradient concentration from 0 to 50 µM or TRAIL WT from 0 to 100 µM were added into each well to a final volume of 150 µL/well. For combination treatment, cells were first treated with DHA, TF, DHA-TF for 30 min. followed with rhTRAIL WT, 4C7, or DHER. After 24 h. incubation, 20 µL [3-(4,5-dimethylthiazol-2-yl)-5-3(carboxymethoxyphenyl0 -2-(4-sulfophenyl)-2H-tetrazolium] MTS reagent was added into each well according to the manufacturer’s instructions (Promega, Leiden, The Netherlands). The absorbance was recorded at 490 nm on a POLARstar Omega microplate reader (Omega, BMG LABTECH GmbH, Ortenberg, Germany).

### Caspase 3/7 Activity Assay

Cells were seeded into white-walled 96-well plates (Greiner bio-one, Alphen a/d Rijn, The Netherlands) and cultured overnight. MDA-MB-231 and MDA-MB-436 were pretreated with DHA, DHA-TF for 30 min., followed with rhTRAIL WT, 4C7, or DHER for 12 h. The Caspase-Glo 3/7 Reagent (Promega Corporation, Madison, WI, USA) was equilibrated to RT, and 50 µL of the reagent was added to each well. The luminescence was collected after the plate was gently mixed and incubated at RT for 1 h.

### Clonogenic Assay

The cells were seeded in 6-well plates at a density of 300,000 cells/well overnight. They were treated with DHA, TF or DHA-TF for 24 h. Then, the treated cells were harvested and seeded in 6-well plates with 400 living cells/well and cultured for 12 days. Finally, the cells were washed with PBS and fixed with methanol for 5 min., then the air-dried plates were stained with Crystal Violet (Sigma-aldrich, St. Louis, USA) and the colonies were counted.

### Apoptotic Assay

The cell apoptosis detection was performed using eBioscience™ Annexin V-FITC/PI Apoptosis Detection Kit (Thermo Fisher Scientific, Carlsbad, CA, USA). After 20 h. treatment, cells were harvested and washed with the binding buffer from the kit. Following the manufacturer’s instructions, cells were incubated with Annexin V-FITC and PI sequentially. Finally, all samples were analyzed by NovoCyte Quanteon Flow Cytometer (Agilent Technologies, California, USA), and 10,000 events were recorded for each analysis.

### ROS Production Assay

The cells were treated with DHA, TF, or DHA-TF adducts for 20 h. Then, all washed and collected samples were incubated with Oxidative Stress Detection Reagent and Superoxide Detection Reagent from ROS-ID^®^ ROS/RNS detection kit (Enzo Life Sciences, Lausen, Switzerland) for 2 h. in the incubator at 37°C and 5% CO_2_. Then the cells were washed and suspended with Wash Buffer. Oxidative stress and Superoxide were detected using a BD FACSCanto™ Flow Cytometry (BD Biosciences, Aalst, Belgium), and 10,000 cells were analyzed for each sample.

### Western Blot Analysis

Cells were harvested after treated with/without DHA-TF for 24h. and western blot was carried out as previously described (25). GPX4 (R&D Systems, Minneapolis, MN) primary antibody was used. Anti-vinculin (Sigma Aldrich, St Louis, USA) was used for confirmation of equal loading of proteins. IRDye^®^ 680RD Goat anti-Mouse IgG secondary antibody was purchased from LI-COR Biosciences (Miami, FL).

### Flow Cytometry of Death Receptors and Transferrin Receptor Expression

Cells were seeded in 6-well plates at a density of 300,000 cells/well overnight. After 24 h. treatment, cells were harvested and washed with FACS buffer (PBS with 2% FBS). Anti-DR4 antibody (Abcam, Cambridge, UK), anti-DR5 antibody (Exbio, Praha, Czech Re-public) or anti-Transferrin antibody was immobilized with cells for 1 h. on ice. The Goat anti-Mouse IgG Alexa Fluor 633 (Thermo Fisher Scientific, Carlsbad, CA, USA) was used as secondary antibody and the mouse IgG Isotype (Dako, Glostrup, Denmark) performed as control.

### Statistical Analysis

Data were presented as a mean ± standard error of the mean (SEM) from three independent experiments. *p* values were analyzed by multiple comparisons test in GraphPad Prism version 8.0 (GraphPad Software, San Diego, CA, USA). * 0.01 < *p* ≤ 0.05, ** 0.001 < *p* ≤ 0.01, *** 0.0001 < *p* ≤ 0.001, **** *p* ≤ 0.0001. All flow cytometry data were analyzed by FlowJo v10.6 (FlowJo, LCC, Oregon, USA).

## Results

### Transferrin Enhances DHA Induced Cell Death in TNBC

**Figure 1:**
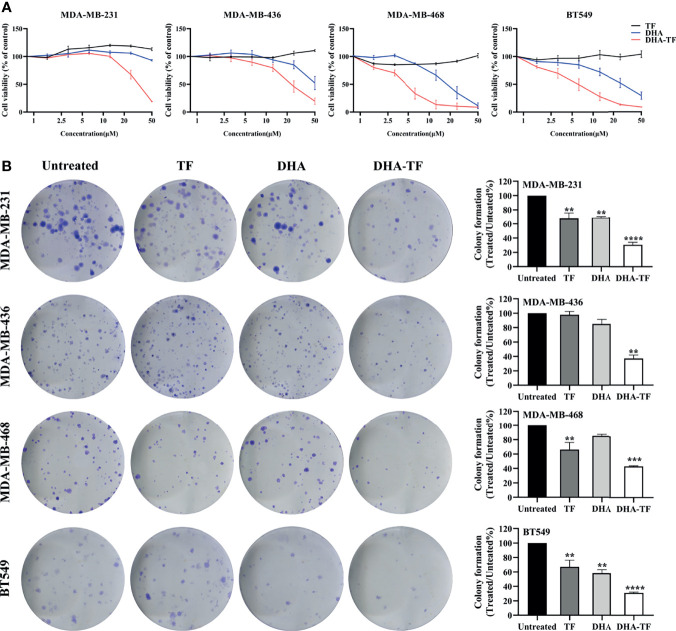
DHA-TF adducts decrease cell viability compared with DHA in TNBC cell lines. **(A)** The sensitivity of MDA-MB-231, MDA-MB-436, MBA-MD-468, and BT549 to TF, DHA, or DHA-TF treatments. The cell viability was evaluated by MTS after cells had been exposed to different treatment options for 24 h. All the results were normalized to the untreated cells which is presented as 100% cell viability. **(B)** Clonogenic assay of DHA-TF for evaluation of long-term cell proliferation. TF, DHA, or DHA-TF was applied to MDA-MB-231 (15 µM), MDA-MB-436 (15 µM), MBA-MD-468 (5 µM), and BT549 (5 µM). Representative images are shown in the left panel, and the quantification of three independent experiments are shown in the right panel as bar graphs. Cells were stained and viable colonies were normalized to the number of colonies in untreated cells. Data shown are mean ± SEM from three independent experiments performed in triplicate. **0.001 < p ≤ 0.01, ***0.0001 < p ≤ 0.001, ****p ≤ 0.0001.

The cell viability of selected TNBC cell lines was recorded after treatment with increasing concentrations of TF, DHA, or DHA-TF adducts by MTS assay (**Figure 1A**). Individual treatment with TF shows no toxicity to all the cell lines (up to 50 µM), while DHA leads to dose-dependent cell death in the range tested (0 µM to 50 µM). Remarkably, the DHA-TF adducts significantly reduced metabolic activity in all cell lines compared with the single TF or DHA treatment. The formation of DHA-TF adducts was checked using fluorescence spectra and the addition of DHA successfully quenches the intrinsic fluorescence of TF at 310 nm in a dose-dependent manner (**Figures S1A, B**). Both DHA and DHA-TF show limited impact at lower concentration (lower than 10 µM) in MDA-MB-231 and MDA-MB-436. These two cell lines are relatively resistant to the individual application of DHA (IC_50_>50 µM), but sensitive to DHA-TF adducts treatment (IC_50 =_ 31.68 and 23.46 µM, respectively). TF especially triggers MDA-MB-468 and BT549 to become more susceptible to DHA induced cell viability reduction (IC_50_ of DHA-TF=4.49 and 5.86 µM, respectively). These cell lines also have a more pronounced response to individual DHA treatment (**Table 1**).

**Table 1:** The IC_50_ of DHA or DHA-TF treatment in different cell lines.

	IC_50_ (µM)	
	DHA	DHA-TF
MDA-MB-231	>50	31.68
MDA-MB-436	>50	23.46
MDA-MB-468	17.80	4.49
BT549	25.72	5.86

The IC_50_ (half-maximal inhibitory concentration) of DHA or DHA-TF treatment in different cell lines was analyzed by Graphpad Prism version 8.0.

This enhancement of cell death under DHA-TF treatment was also observed in clonogenic assays. **Figure 1B** shows that DHA-TF results in a significant reduction of colony numbers across all cell lines, which matches the MTS results. Moreover, colonies with DHA-TF challenges are generally smaller in size than untreated or other treated cells (**Figure 1B**, left panel). The statistical analysis from three independent experiments confirmed that the effects of DHA-TF adducts in reducing colony numbers is reproducible (**Figure 1B**, right panel). Meanwhile, TF or DHA can also inhibit colony formation to a certain extent in comparison with untreated cells in MDA-MB-231, MDA-MB-468 and BT549 cells.

### DHA-TF Adducts Generates ROS and Superoxide in TNBC

**Figure 2:**
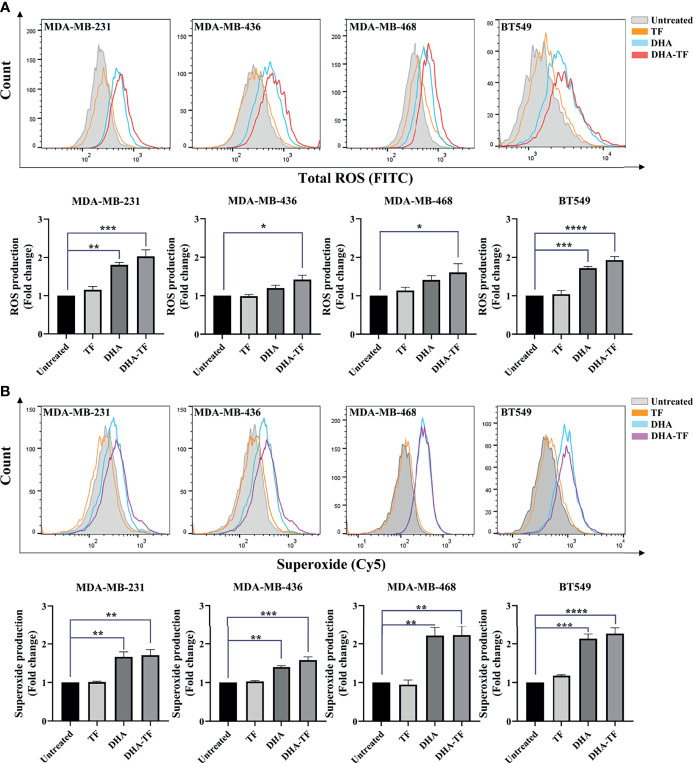
ROS and superoxide are produced by DHA and DHA-TF in TNBC cells. Total ROS **(A)** and superoxide **(B)** production were detected in MDA-MB-231, MDA-MB-436, MBA-MD-468, and BT549 cells under different treatment for 20 h. Results are quantified as fold changes of ROS or superoxide production under the treatments relative to untreated group in bar graphs. Data shown are mean ± SEM from three independent experiments performed in triplicate. *0.01 < p ≤ 0.05, **0.001 < p ≤ 0.01, ***0.0001 < p ≤ 0.001, ****p ≤ 0.0001.

Artemisinins have been reported to generate ROS production in cancer cells and parasites. We performed the ROS detection assay in TF, DHA, or DHA-TF treated TNBC cells to understand the potency of these treatments in generating ROS. As seen in all the samples, TF treatment shows almost no impact on total ROS production compared with untreated ones (**Figure 2A**). In both DHA and DHA-TF treated cells, more total ROS were generated and the most substantial increase was evident from the DHA-TF treatment in all cell lines. Furthermore, we found increased superoxide production in DHA or DHA-TF treated cells, which is the essential ROS species responsible for lipid peroxidation and membrane destabilization (28). In summary, TF has negligible effects on both total ROS and superoxide generation, whereas DHA and DHA-TF significantly increase their production in all tested cell lines (**Figure 2B**).

### DHA-TF Adducts Induce Cell Apoptosis and Ferroptosis in TNBC

**Figure 3:**
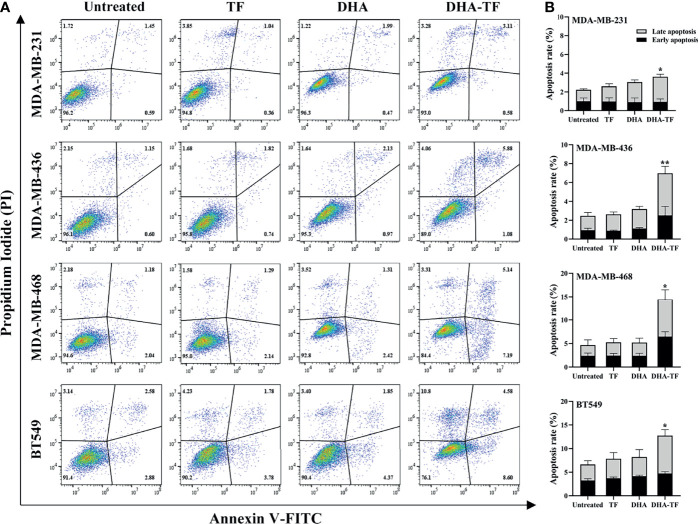
DHA-TF adducts induce cell apoposis in TNBC cell lines. **(A)** TF, DHA and DHA-TF adducts were added to MDA-MB-231 (15 µM), MDA-MB-436 (15 µM), MBA-MD-468 (5 µM), and BT549 (5 µM) for 20 h. **(B)** Statistical analysis was based on three independent experiments. Apoptosis assay was performed by flow cytometry using annexin V/PI staining, and data were analyzed by FlowJo V10. *p* values were compared to untreated control. *0.01 < p ≤ 0.05, **0.001 < p ≤ 0.01.

Above, we proved that DHA-TF adducts significantly reduce cell viability and clonogenic capacity in TNBC cells. It has been established that artemisinins induce cell apoptosis in colon cancer cells (25). Thus, cell apoptosis of the DHA-TF treated TNBC cells was analyzed by Annexin V/PI assay and a significant increase was observed in all four cell lines compared with the untreated cells (**Figure 3A**). The DHA-TF adducts induce 4% and 7% cell apoptosis in MDA-MB-231 and MDA-MB-436 respectively. Whereas more cell apoptosis was observed in MDA-MB-468 (15%) and BT549 (13%), indicating a higher sensitivity to DHA-TF treatment (**Figure 3B**).

**Figure 4:**
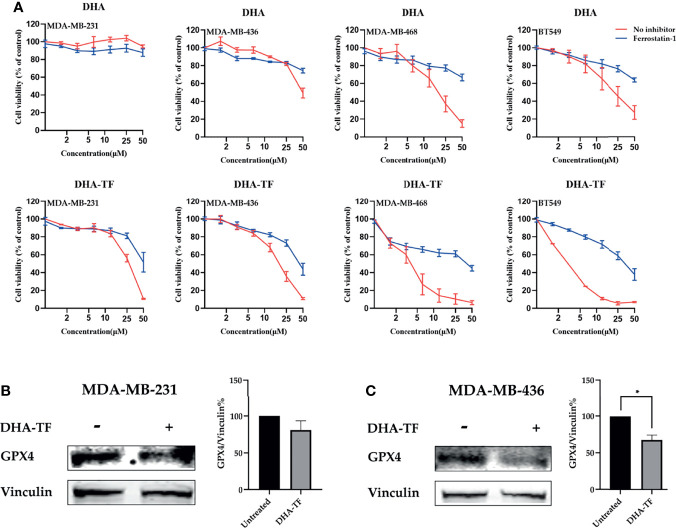
DHA-TF adducts induce ferroptosis in TNBC cell lines. **(A)** Cells were treated with ferrostatin-1, followed with increasing concentration of DHA or DHA-TF in each cell line for 24 h. Data shown are mean ± SEM from three independent experiments performed in triplicate. The GPX4 expression was analyzed in **(B)** MDA-MB-231 and **(C)** MDA-MB-436 after treatment with/without 15 μM DHA-TF. The relative GPX4 expression was calculated based on integrated density and shown in bar chart. The Date shown are mean ± SEM from duplicate independent experiments. *0.01 < p ≤ 0.05.

In the follow-up experiment, the cells were pretreated with the ferroptosis inhibitor ferrostatin-1 (Fer-1), followed by increasing concentrations of DHA or DHA-TF (**Figure 4A**). Similar as described above, both DHA and DHA-TF reduce cell viability, and DHA-TF is more effective than DHA alone. Interestingly, the ferroptosis inhibitor Fer-1 partially rescues all the cell lines from the DHA or DHA-TF induced cell death. MDA-MB-468 and BT549 are most susceptible to DHA-TF treatment, and Fer-1 significantly reduces their cell death. This indicates that the cell death triggered by both DHA and DHA-TF in all TNBC cell lines is partially caused by ferroptosis. We also analyzed the expression of glutathione peroxidase 4 (GPX4) (**Figures 4B, C**), a crucial anti-oxidative enzyme, in MDA-MB-231 and MDA-MB-436. GPX4 is significantly downregulated in MDA-MB-436 after DHA-TF treatment, indicating that DHA-TF induced ferroptosis through suppressing GPX4 expression.

### DHA-TF Enhances DHER-Induced Apoptosis in MDA-MB-231 and MDA-MB-436

**Figure 5:**
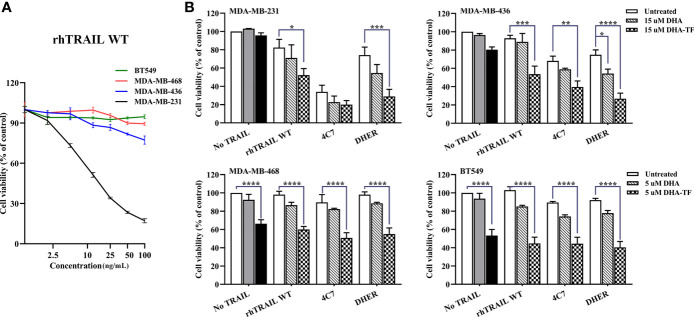
DHA-TF adducts enhance TRAIL efficacy in TRAIL-sensitive TNBC cell lines. **(A)** The sensitivity of TNBC cell lines to TRAIL-induced apoptosis. All cell lines were treated with different concentration of rhTRAIL WT for 24 h. **(B)** All cell lines were pre-treated with DHA or DHA-TF for 30 min, followed with 5 ng/mL rhTRAIL WT, 4C7, or DHER for 24 h. Individual treatment of DHA is shown in grey, and individual treatment of DHA-TF is shown in black. Cell viability was determined by MTS assay. Data shown are mean ± SEM from three independent experiments performed in triplicate. *0.01 < p ≤ 0.05, **0.001 < p ≤ 0.01, ***0.0001 < p ≤ 0.001, ****p ≤ 0.0001.

To enlarge the therapeutic window of DHA-TF in TNBC, we combined it with TRAIL variants to evaluate their efficacy in inducing cell apoptosis. We treated all the cells with wildtype (WT) recombinant human TRAIL (rhTRAIL) and the cell viability was determined. After 24 h incubation with increasing concentration of rhTRAIL, MDA-MB-231 is highly sensitive, MDA-MB-436 is slightly sensitive, MDA-MB-468 and BT549 are resistant to TRAIL-induced apoptosis (**Figure 5A**).

Then, we combined DHA or DHA-TF with rhTRAIL WT, DR4-specific TRAIL (4C7) or DR5-specific TRAIL (DHER) to understand their influence on TRAIL pathway. In **Figure 5B**, DHA-TF significantly enhances rhTRAIL WT and DHER cytotoxicity compared to individual TRAIL treatment (white bar) or single DHA-TF treatment (black bar) in MDA-MB-231 and MDA-MB-436. A dose-dependent cell viability reduction upon combining DHA-TF with TRAIL WT or DHER is observed especially in MDA-MB-436 (**Figure S2**). On the other hand, the addition of TRAIL shows little extra cell death in MDA-MB-468 and BT549. The individual DHA-TF already presents a considerable cancer cell lethality (black bar). Thus, the combination of DHA-TF with TRAIL is a promising treatment to the cells which are more susceptible to TRAIL, such as MDA-MB-231 and MDA-MB-436.

**Figure 6:**
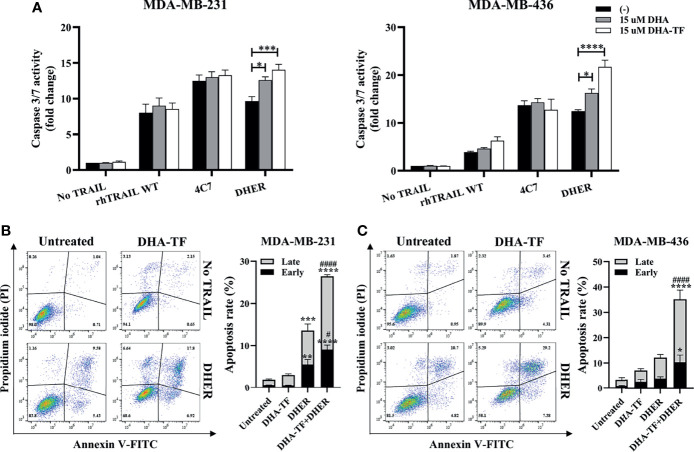
DHA-TF enhances DHER-induced apoptosis through caspase cascade. **(A)** DHA-TF in combination with DHER significantly enhances caspase3/7 activity in MDA-MB-231 and MDA-MB-436. Luminescence was measured after 1 h reagent incubation. Data shown are mean ± SEM from three independent experiments performed in triplicate. The cell apoptotic assays were performed in MDA-MB-231 **(B)** and MDA-MB-436 **(C)** after 20 h treatment. Statistical analysis was based on three independent experiments and is shown on the right side. In **(B, C)**, the * represents the comparison with untreated cells, and the # represents the comparison with DHER treated cells. *0.01 < p ≤ 0.05, **0.001 < p ≤ 0.01, ***0.0001 < p ≤ 0.001, ****p ≤ 0.0001, ^#^0.01   p ≤ 0.05, ^####^p ≤ 0.0001..

In order to confirm whether the cell viability reduction is from the TRAIL-induced apoptosis pathway, caspase 3/7 activity was measured in MDA-MB-231 and MDA-MB-436 (**Figure 6A**). Individual DHA or DHA-TF treatments show limited effects on caspase 3/7 activation in both cell lines, while a minor effect is observed with rhTRAIL WT or 4C7. Most significantly, both DHA and DHA-TF in combination with DHER strongly improve the caspase activation in both cell lines tested, and DHA-TF establishes the best response. Because of the increases in caspase 3/7 activity, we analyzed the effects in early and late stages apoptosis in the DHA-TF and DHER treated cells (**Figures 6B, C**). Whereas DHA-TF alone shows limited effects on the cells, the individual DHER significantly induces cell apoptosis in MDA-MB-231. Importantly, DHA-TF remarkably enlarges DHER-induced apoptosis compared to untreated cells (*p* ≤ 0.0001) or to DHER treated cells (*p* ≤ 0.0001) in both cell lines.

### DHA-TF Induces DR5 Expression on TNBC Cell Surface

**Figure 7:**
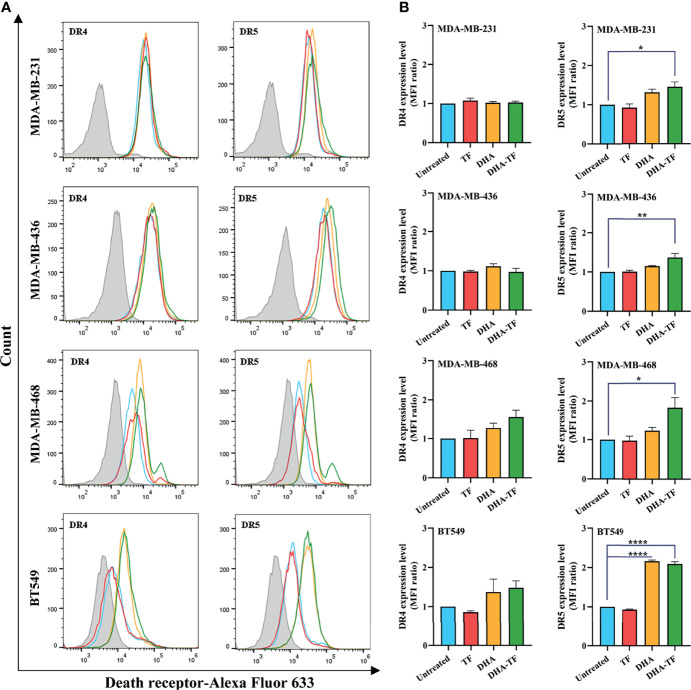
DHA-TF significantly upregulates DR5 expression on the surface of TNBC cells. **(A)** TF, DHA, or DHA-TF were added to MDA-MB-231 (15 µM), MDA-MB-436 (15 µM), MBA-MD-468 (5 µM), and BT549 (5 µM) for 24 h. The histograms of death receptors expression were analyzed using FlowJo v10 software. **(B)** The mean fluorescence intensity (MFI) ratio relative to unstained cells (filled grey peaks) is shown in bar graphs. Data shown are mean ± SEM from three independent experiments. *0.01 < p ≤ 0.05, **0.001 < p ≤ 0.01, ****p ≤ 0.0001.

Judging from the different performance of rhTRAIL WT, 4C7, and DHER, it might be speculated that DHA-TF affects the expression of different death receptors in TNBC cells. Therefore, we analyzed the expression of DR4 and DR5 on the cell surface after TF, DHA, or DHA-TF treatment. Firstly, compared to the unstained cells (filled grey), the peak of the untreated cells (open blue) shifted to the right, indicating that all the cells have DR4 and DR5 expression on their surface (**Figure 7A**). Then, upon TF treatment (open red), no influence on DR4 and DR5 expression compared with untreated cells can be seen. Also, there is no change in DR4 expression due to DHA or DHA-TF in MDA-MB-231 or MDA-MB-436, although slight enhancement is observed in MDA-MB-468 or BT549 cells (not significant) (**Figure 7B**). On the other hand, DHA-TF significantly upregulates DR5 expression in all the cell lines with the following changes of the MFI ratio: MDA-MB231 (1.5-fold), MDA-MB-436 (1.4-fold), MDA-MB-468 (1.8-fold), and BT549 (2.1-fold). As another control, none of the treatments interfere with the expression of transferrin receptor in the tested TNBC cells (**Figure S3**).

### DHA-TF Stimulated DHER-Induced Apoptosis Is P53-Independent and ROS-Dependent in TNBC

**Figure 8:**
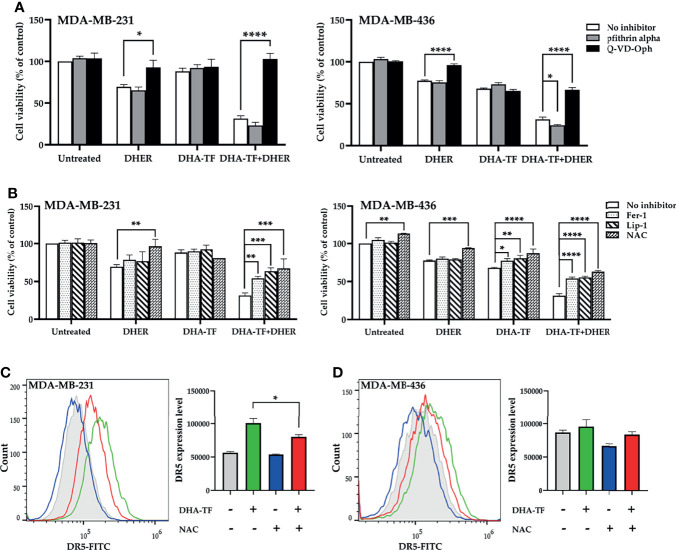
Caspase inhibitor and ROS inhibitor rescue cells from DHA-TF combined DHER-induced cell death. **(A)** Cells were pretreated with/without 20 µM pfithrin alpha or 10 µM Q-VD-Oph for 2 h, followed with 15 µM DHA-TF and/or 5 ng/mL DHER. **(B)** Instead of pfithrin alpha and Q-VD-Oph, the cells were pretreated with 10 µM ferrostatin-1, 2 µM liproxstatin-1, or 5 mM N-Acetyl-L-cysteine. Cell viability was determined by MTS assay. NAC decreases DR5 expression in DHA-TF treated MDA-MB-231 **(C)** and MDA-MB-436 **(D)** cells. The geometric mean of DR5 expression relative to isotype is analyzed using FlowJo v10 software and shown in bar graphs. Data shown are mean ± SEM from three independent experiments. *0.01 < p ≤ 0.05, **0.001 < p ≤ 0.01, ***0.0001 < p ≤ 0.001, ****p ≤ 0.0001.

DHA-TF adducts increase DR5 expression and enhance DHER-induced apoptosis in MDA-MB-231 and MDA-MB-436. We previously reported that DHA stimulates P53-mediated DR5 upregulation in colon cancer cell line HCT116. Different from HCT116, all tested TNBC cell lines harbor mutated P53. Thus, we analyzed the influence of P53 inhibitor pfithrin alpha on DHA-TF treated MDA-MB-231 and MDA-MB-436 (**Figure 8A**). In MDA-MB-231, DHA-TF combined with DHER reduces the cell viability to 31%, while the addition of pfithrin alpha leads to a similar cell lethality. In contrast, the caspase inhibitor Q-VD-Oph successfully rescues the cells. Comparable results were observed in MDA-MB-436. In addition, Q-VD-Oph also rescues both cells from individual DHER-induced apoptosis.

Apart from P53 and caspase inhibitors, several ROS inhibitors [N-Acetyl-L-cysteine (NAC), ferrostatin-1 (Fer-1), and liproxstatin-1(Lip-1)] were used to eliminate the effects from the combination treatment. All the tested ROS inhibitors significantly increase the cell viability in the DHA-TF and DHER treated cells (**Figure 8B**). We also found that these ROS inhibitors rescued MDA-MB-436 from DHA-TF-induced cell death. This did not occur in MDA-MB-231, which can be explained by its lower sensitivity to DHA-TF. Afterwards, the DR5 expression was analyzed in the cells which were pretreated with NAC followed with DHA-TF (**Figures 8C, D**). As we presented above, DHA-TF increases cell surface DR5 expression, which can be significantly down-regulated by NAC in MDA-MB-231. The same pattern was observed in MDA-MB-436. In summary, ROS production triggers DR5 expression in the DHA-TF treated cells, making the cells more susceptible for DHER treatment.

## Discussion

In 2020, approximately 531,068 new cases were diagnosed with breast cancer in Europe, and 141,765 people of both sexes died, according to Globocan 2020. As the most aggressive breast cancer subtype with high mortality and poor prognosis, TNBC’s metastasis leads to less than two years median overall survival time, which is substantially shorter than the other subtypes (29). For treating TNBC, the apoptosis-inducing agent TRAIL has been extensively investigated due to its tumor-specific properties (30). To enhance TRAIL-induced apoptosis in TNBC, various natural products have been proved effective, such as pterostilbene and ursolic acid (31, 32). However, most of them are still under clinical trials (31, 33). In order to accelerate clinical trials, dihydroartemisinin, World Health Organization (WHO)-approved drug, was used as a combination therapy with TRAIL in cancer cells (34). At the same time, transferrin was also used to further improve the efficacy of DHA by the formation of the protein-drug adducts.

It has been proven that DHA binds to TF mainly through van der Waals forces and hydrogen bonds, and the molar binding ratio is 1:1. Furthermore, it is important to form the DHA-TF adducts in serum-free medium to avoid the formation of DHA-BSA (15). The quenching of intrinsic fluorescence indicates that DHA changes the microenvironment of chromophores and binds to TF (**Figure S1B**), which confirms that DHA interacts with TF at neutral pH (17). Normally, artemisinins enter the cells through passive diffusion, which is uncontrolled, slow and inefficient (35). By forming the DHA-TF adducts, these will be delivered into cells *via* TfR-mediated endocytosis. On one hand, higher TfR expression on the cancer cell surface makes it a promising method of delivering DHA to tumors (36). On the other hand, more iron transported into cells through transferrin increased DHA-derived ROS production and induced more cell death (37).

In the present paper, we demonstrated that DHA-TF decreases cell viability and produces more ROS/superoxide compared with DHA in TNBC (**Figures 1** and **2**). Interestingly, DHA-TF adducts exhibited different effects in the tested cell lines, which does not correspond to their TfR cell surface expression level. Similar findings were reported previously in the human prostate carcinoma cell line DU 145 (38). Compared between cell lines, both MDA-MB-468 and BT-549 are more susceptible to DHA treatment, making them more sensitive to DHA-TF (**Table 1**).

It has been reported that DHA or DHA-TF activates caspase-9 and induces cell apoptosis *via* the intrinsic pathway (38, 39). Here, we found that DHA-TF also stimulates DR5 expression on TNBC cell surface (**Figure 7**), suggesting the possibility to induce cell apoptosis through the extrinsic pathway with the combination with TRAIL. What’s more, with the DR5-specific TRAIL variant DHER, the cell viability in MDA-MB-231 and MDA-MB-436 is significantly decreased (**Figures 5** and **6**). Even though DHA-TF successfully increases DR5 expression in all tested TNBC cell lines, the TRAIL resistant feature of MDA-MB-468 and BT-549 limits the efficacy of the combination treatment. It is reported that the downregulation of c-FLIP or XIAP expression enhances TRAIL sensitivity in MDA-MB-468, which could be implemented in the near future (40, 41). Besides, we only analyzed the effectiveness of the combination treatment in cultured cells, the specificity of the combination to tumor cells *in vivo* still needs further investigation. DHA-TF or TRAIL loaded nanoparticles have been proved to be effective and specific to tumor cells *in vivo* (42–44), Which will be a promising delivery method for the combination treatment for further analysis.

**Figure 9:**
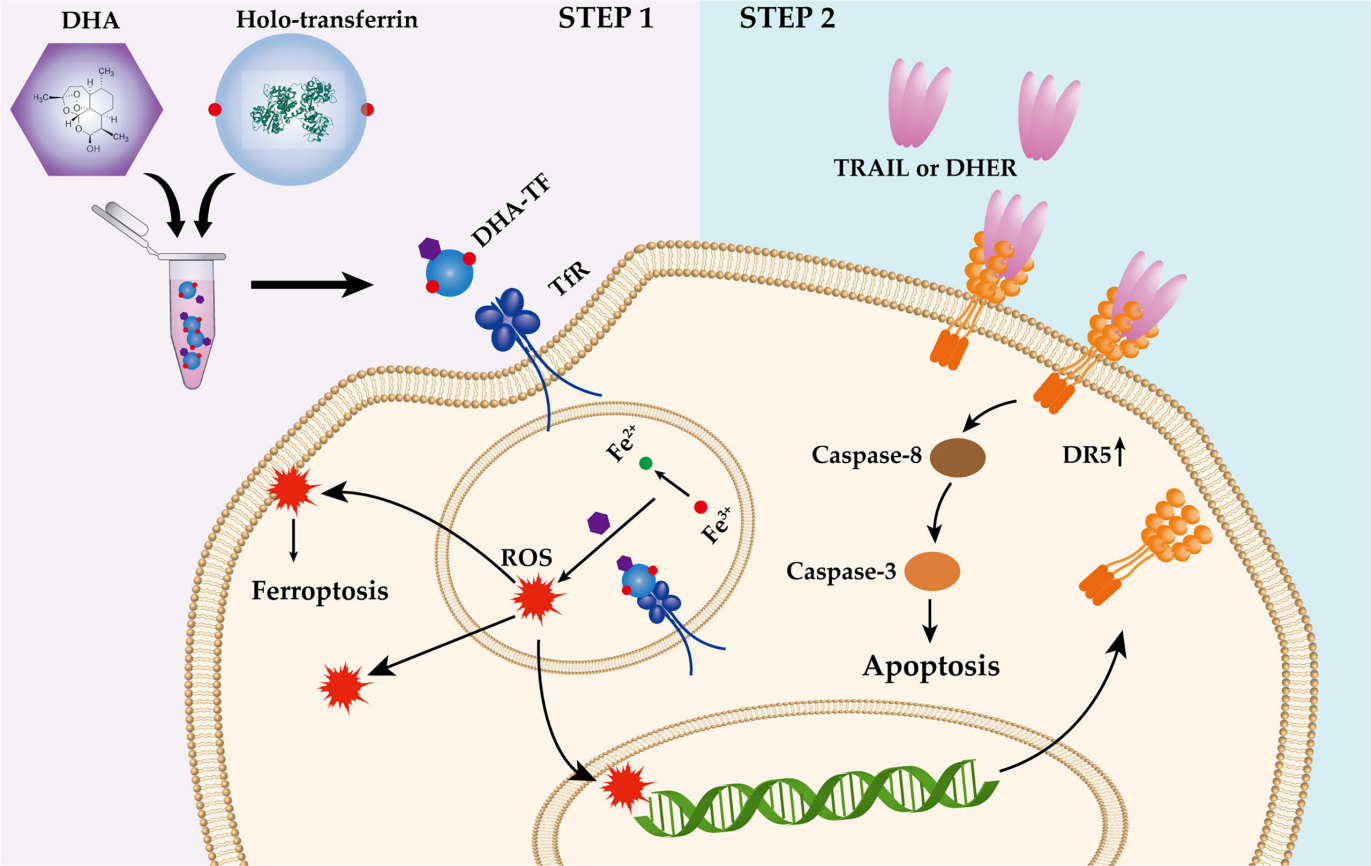
Schematic illustration of the DHA-TF with DHER treatment in TNBC cells.

The P53 status is important for artemisinin induced DR5 upregulation (25). However, most of the TNBC cell lines contain mutated P53 (45), for example, MDA-MB-231 (R280K) (46), MDA-MB-436 (E204 frameshift to stop codon) (47), MDA-MB-468 (R273H) (47) and BT-549 (R249S) (48). All the mentioned mutations are located on the P53 DNA binding site, interfering the regulation of P53 to its target genes, such as DR5 (49). Here, we found that DHA induces DR5 expression in a P53-independent and ROS-dependent manner (**Figure 8A**). It has been reported that the ART-type drugs are able to induce endoplasmic reticulum (ER) stress response and upregulate the CCAAT/-enhancer-binding protein homologous protein (CHOP) expression in various tumor cells (7, 50, 51). What’s more, we and other groups have demonstrated that CHOP is responsible for the DR5 upregulation under certain stresses (52–54). Therefore, it is of interest to investigate the influence of DHA-TF on the expression of CHOP, and the linkage between CHOP and DR5 expression under DHA-TF stress. Based on our presented data, we visualized the combination treatment of DHA-TF with DHER in the TNBC cells in a schematic illustration (**Figure 9**). DHA was incubated with TF in serum-free medium to form DHA-TF adducts. Then, the mixture was added to TNBC cells. Holo-transferrin transports iron and DHA into cells through transferrin receptor-mediated endocytosis. With the decreased pH in the endosome, DHA is released from TF. At the same time, ferrireductase reduces Fe^3+^ to Fe^2+^ (55), which converts DHA into ROS by cleaving the endoperoxide bridge. The accumulated ROS induces apoptosis, ferroptosis and upregulates DR5 expression. Then, TRAIL or DHER was added into the DHA-TF pretreated TNBC cells and significantly increases cell apoptosis.

In summary, DHA-TF adducts induce more cell death in TNBC cells by triggering both the apoptosis and ferroptosis signaling pathways. In addition, this treatment increases DR5 expression on the cell surface, making the cells more susceptible to the DR5-specific TRAIL variant. Furthermore, DR5 upregulation is P53-independent and ROS-dependent in the TNBC cells. Our data showed that the combination of DHA-TF with DHER can be a potent strategy for TNBC patients in clinical trials.

## Data Availability Statement

The original contributions presented in the study are included in the article/**Supplementary Material**. Further inquiries can be directed to the corresponding author.

## Author Contributions

XZ, AS-G, and FN designed and performed the experiments. XZ and AS-G analysed data. XZ wrote the original draft. XZ, AS-G, RS, and WQ reviewed the manuscript. WQ supervised the research and acquired funding. All authors contributed to the article and approved the submitted version.

## Funding

Financial support was received in the form of the scholarship from China Scholarship Council (CSC) for XZ (201708610140). WQ received funding from the Stichting voor de Technische Wetenschappen (STW), grant 11056.

## Conflict of Interest

The authors declare that the research was conducted in the absence of any commercial or financial relationships that could be construed as a potential conflict of interest.

## Publisher’s Note

All claims expressed in this article are solely those of the authors and do not necessarily represent those of their affiliated organizations, or those of the publisher, the editors and the reviewers. Any product that may be evaluated in this article, or claim that may be made by its manufacturer, is not guaranteed or endorsed by the publisher.
