# Supplementary material for: Dihydroartemisinin-Transferrin Adducts Enhance TRAIL-Induced Apoptosis in Triple-Negative Breast Cancer in a P53-Independent and ROS-Dependent Manner
Source: Front Oncol. 2022 Jan 3;11:789336. doi: 10.3389/fonc.2021.789336 (PMC8762273; doi:10.3389/fonc.2021.789336)
Supplement: Supplementary file 1 [file DataSheet_1.pdf]

Dihydroartemisinin-transferrin adducts enhance TRAIL-induced apoptosis in triple-negative breast cancer

Xinyu Zhou[1], Abel Soto-Gamez[1 2], Fleur Nijdam[1], Rita Setroikromo[1], Wim J Quax[1*]

[1] Department of Chemical and Pharmaceutical Biology, Groningen Research Institute of Pharmacy, University of Groningen, Groningen, Netherlands

[2] European Institute for the Biology of Aging (ERIBA), University Medical Center Groningen (UMCG), Groningen, Netherlands.

Supplementary Material

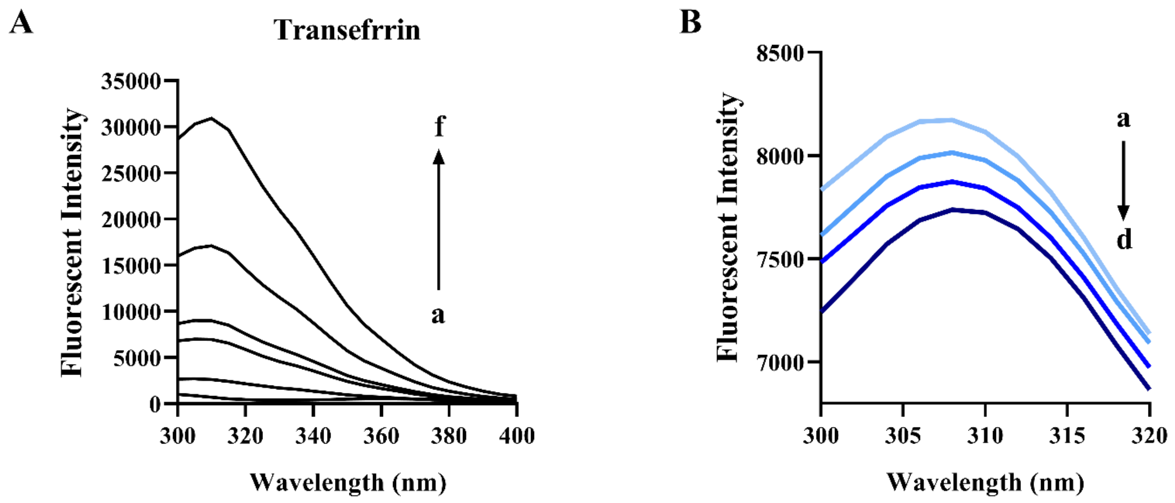

Supplementary Figure 1. DHA quenches intrinsic fluorescence of transferrin (λex=270 nm). (A) The intrinsic fluorescence of transferrin at different concentrations was measured, and the peak was recorded at 310 nm. Curves from (a) to (f) represent 0, 1, 3, 5, 10, 30 µM of TF, respectively. (B) The intrinsic fluorescence of 10 µM TF was quenched with an increasing concentration of DHA. Curves from (a) to (d) represent 0, 1, 5, 10 µM of DHA, respectively.

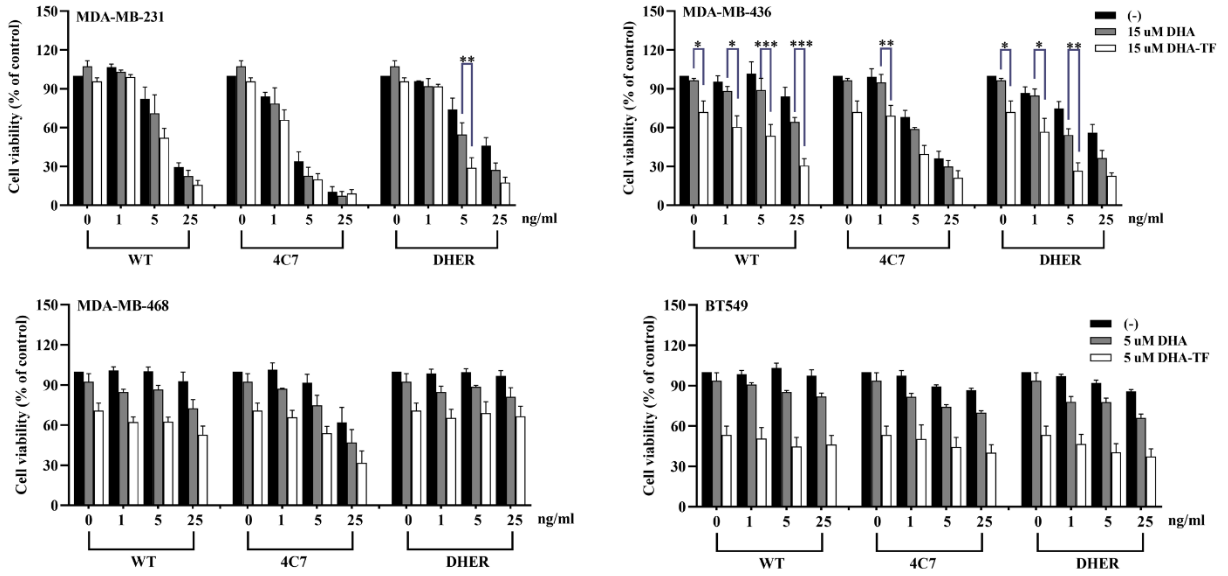

Supplementary Figure 2. Combination treatment of DHA/DHA-TF with different concentration of TRAIL variants. All cell lines were pre-treated with DHA or DHA-TF for 30 min, followed with 0, 1, 5, 25 ng/mL rhTRAIL WT, 4C7, or DHER for 24 h. Cell viability was determined by MTS assay. Data shown are mean ± SEM from three independent experiments performed in triplicate.

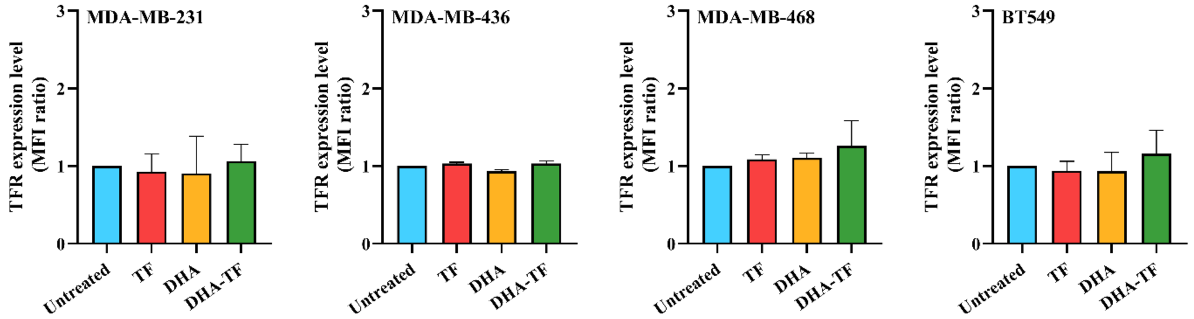

Supplementary Figure 3. Transferrin receptor expression on TNBC cells after treated with TF, DHA, or DHA-TF. TF, DHA, or DHA-TF were applied to MDA-MB-231 (15 µM), MDA-MB-436 (15 µM), MBA-MD-468 (5 µM), and BT549 (5 µM) for 24 h. The mean fluorescence intensity (MFI) ratio is relative to unstained cells with mean ± SEM from three independent experiments.
